# Retraction: eliciting broadly neutralizing antibodies against HIV-1 that target gp41 MPER

**DOI:** 10.1186/1742-4690-11-16

**Published:** 2014-02-06

**Authors:** D Han, H Habte, Y Qin, K Takamoto, C LaBranche, D Montefiori, MW Cho

**Affiliations:** 1 Albert Einstein College of Medicine, 1300 Morris Park Avenue, Bronx, NY 10461, USA; 2 Duke University, Durham, NC 27708, USA; 3 Iowa State University, Ames, IA 50011, USA

## Retraction

This poster presentation [1] has been retracted on request of the author Michael W Cho. The Office of Research Integrity at the US DHHS has determined that the first author Dong-Pyou Han committed research misconduct and the poster was prepared based on falsified data [2].
